# Large-Scale Biogeographical Shifts of Abundance of Antibiotic Resistance Genes and Marine Bacterial Communities as Their Carriers along a Trophic Gradient

**DOI:** 10.3390/ijms25010654

**Published:** 2024-01-04

**Authors:** Mia Dželalija, Željana Fredotović, Nikolina Udiković-Kolić, Hrvoje Kalinić, Slaven Jozić, Ivica Šamanić, Marin Ordulj, Ana Maravić

**Affiliations:** 1 Department of Biology, Faculty of Science, University of Split, 21000 Split, Croatia; mdzelalij@pmfst.hr (M.D.); zfredotov@pmfst.hr (Ž.F.); isamanic@pmfst.hr (I.Š.); 2 Division for Marine and Environmental Research, Ruđer Bošković Institute, 10002 Zagreb, Croatia; nudikov@irb.hr; 3 Department of Informatics, Faculty of Science, University of Split, 21000 Split, Croatia; hkalinic@live.pmfst.hr; 4 Institute of Oceanography and Fisheries, 21000 Split, Croatia; sjozic@izor.hr; 5 University Department of Marine Studies, University of Split, 21000 Split, Croatia; marin.ordulj@unist.hr

**Keywords:** Adriatic Sea, antibiotic resistance, bottom layer, environmental factors, marine environment, Mediterranean, seasonal and spatial distribution, seawater, surface water, qPCR

## Abstract

The role of marine environments in the global spread of antibiotic resistance still remains poorly understood, leaving gaps in the One Health-based research framework. Antibiotic resistance genes (ARGs) encoding resistance to five major antibiotic classes, including sulfonamides (*sul1*, *sul2*), tetracyclines (*tetA*, *tetB*), β-lactams (*bla*_CTX-M_, *bla*_TEM_ *bla*_VIM_), macrolides (*ermB*, *mphA*), aminoglycosides (*aac3-2*), and integrase gene (*intl1*) were quantified by RT-qPCR, and their distribution was investigated in relation to environmental parameters and the total bacterial community in bottom layer and surface waters of the central Adriatic (Mediterranean), over a 68 km line from the wastewater-impacted estuary to coastal and pristine open sea. Seasonal changes (higher in winter) were observed for antibiotic resistance frequency and the relative abundances of ARGs, which were generally higher in eutrophic coastal areas. In particular, *intl1*, followed by *bla*_TEM_ and *bla*_VIM_, were strongly associated with anthropogenic influence and *Gammaproteobacteria* as their predominant carriers. Water column stratification and geographic location had a significant influence on ARGs distribution in the oligotrophic zone, where the bacterial community exhibited a seasonal shift from *Gammaproteobacteria* in winter to Marine group II in summer.

## 1. Introduction

The escalating levels of antibiotic resistance in human pathogens have caused a global public health emergency and raised significant apprehension [1]. Aquatic ecosystems are among the natural environments of the highest concern when it comes to the research of antibiotic resistance phenomenon within the One Health framework [2]. Namely, the coastal area is a highly diverse and densely populated ecosystem where the terrestrial environment overlaps with the marine, making it important but challenging to study the transmission pathways of resistance determinants.

Previous studies have shown that antibiotic resistance genes (ARGs) can be very widespread and diverse in the marine environment, especially in coastal areas [3,4]. These genes can originate from a variety of sources, including human-impacted rivers [5], submarine outfalls from wastewater treatment plants [6], as well as direct contamination from recreational water users [7]. However, much remains to be understood about the fate of ARGs and their bacterial reservoirs in the marine environment, particularly with regard to their spatial and seasonal nature. It appears that the dynamics of ARGs are complex and influenced by multiple factors. Studies have shown that shifts in bacterial communities [8] and environmental conditions such as nitrogen and phosphorus concentrations, pH, salinity, and temperature play an important role in the accumulation of ARGs [9,10,11,12]. It is, therefore, crucial to investigate the comprehensive profiles of ARGs, as well as the main factors behind their spread.

In our previous study, we provided extensive taxonomic and functional profiles of the marine microbial communities obtained seasonally from bottom and surface waters in the central Adriatic (Mediterranean), covering a 68 km line from the wastewater-influenced river mouth to coastal and pristine open sea [8]. We found that the resistome was more diversified along the trophic gradient in winter, but cationic antimicrobial peptide (CAMP), vancomycin, and multidrug resistance modules prevailed regardless of trophic status or season. Nonetheless, resistance determinants for multidrug, beta-lactams (particularly imipenem), macrolides, aminoglycosides, and phenicols were much more common in winter, indicating that the diversity of native resistomes is greatly contingent on seasonal variations in the water column, caused by thermohaline stratification and nutrient availability [8].

In the present study, we aim to (i) quantify by RT-qPCR the clinically relevant ARGs associated with resistance to five major classes of antibiotics, including two sulfonamide resistance genes (*sul1*, *sul2*), two tetracycline resistance genes (*tetA*, *tetB*), three β-lactam resistance genes (*bla*_CTX-M_, *bla*_TEM_, *bla*_VIM_), two macrolide resistance genes (*ermB*, *mphA*), one aminoglycoside resistance gene (*aac3-2*), and the class 1 integron integrase gene (*intl1*); (ii) determine how environmental factors, including physico-chemical and biological factors, influence the abundance of these genes along the trophic gradient; (iii) associate specific bacterial taxa with targeted ARGs to gain deeper insights into gene distribution and resistome structure in the marine environment.

## 2. Results

### 2.1. Frequency of Antibiotic Resistance along the Trophic Gradient

**Figure 1:**
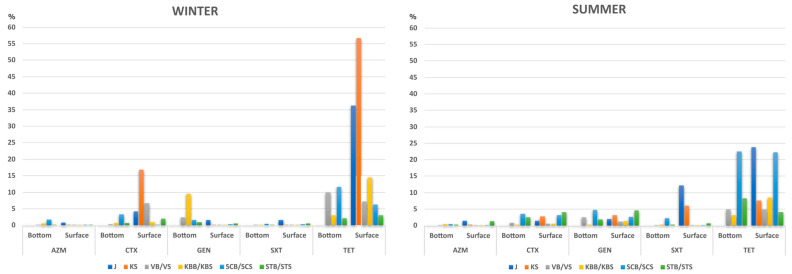
Frequency of resistance to five major classes of antibiotics in summer and winter along the trophic gradient encompassing eutrophic (JS, KS), mesotrophic (VBS/VBB, KBS/KBB), and oligotrophic (SCS/SCB, STS/STB) marine areas. The percentage was calculated as the ratio between the number of bacteria grown on agar infused with antibiotics per mL and the total heterotrophic bacteria.

The proportion of antibiotic-resistant heterotrophic bacteria in the surface and bottom layers along the trophic gradient was evaluated against antimicrobial agents belonging to five major antibiotic classes, including macrolides, tetracyclines, aminoglycosides, β-lactams, and sulfonamides. In most cases, a higher percentage of antibiotic-resistant bacteria was detected in winter than in summer, although these rates differed seasonally along the trophic gradient (Figure 1; Table S2). These differences were statistically significant at open-sea oligotrophic site STS/STB (paired *t*-test, *p* = 0.02) and were primarily related to cefotaxime—(CTX)-resistant bacteria (2.5% of resistant bacteria at STB and 4.03% at STS in summer, compared to 0.7% and 2.0% at the same site in winter). In addition, tetracycline—(TET)-resistant bacteria also greatly contributed to these shifts at open sea; 8.23% and 4.03% of resistant bacteria were recorded in summer, compared to 2.12% and 3.1% in winter, respectively.

Gentamicin (GEN) resistance was found to be of particular importance for the bacterial community in the surface layer and led to the largest rate shifts in summer (paired *t*-test, *p* < 0.01). This was most evident at STS (4.6% in summer and 0.63% in winter) and KS (3.16% in summer and 0.02% in winter). In comparison, more bacteria resistant to GEN were found at the bottom layer in winter (up to 9.6% in KBB), and this was less seasonally influenced (Table S2).

In contrast to GEN, TET resistance in surface waters was among the highest measured regardless of season; it followed the trophic gradient and decreased from the coast to the open sea. Namely, in the winter, it ranged from 56.7% and 36.3% at the eutrophic sites KS and J to 3.1% at the oligotrophic STS, while in summer, the values at the same sites were 7.6%, 23.8%, and 4.03% (Figure 1; Table S2).

Moreover, the highest sulfamethoxazole (SXT) resistance was recorded in the surface layer in summer, and it followed the trophic gradient regardless of season (from 12.14% and 6.03% at J and KS to 0.7% at STS in summer, with 1.67%, 0.01%, and 0.63% of SXT resistance at the same sites in winter) (Figure 1; Table S1). Seasonality also had no effect on the rate of resistance to this antibiotic in the bottom layer; however, this community appeared to be site-specific rather than driven by trophic status.

The percentage of CTX-resistant bacteria was found to be seasonal and highest in winter, while at the same time, it followed the trophic gradient and was more pronounced in the surface layer microbial community (16.81%, 6.73%, and 3.33% at KS, VS, and STS, respectively) (Figure 1).

Furthermore, we found that azithromycin (AZM) resistance in the surface bacterial community was not dependant on the seasonality but rather on trophic status. However, the bottom layer community exhibited similar rates from the nearshore to the open sea in summer (approximately 0.4% resistance from KBB to STB), whereas this pattern was somewhat different in the winter season, showing variations from 0.1% to 1.8% along the trophic gradient (Figure 1; Table S2).

### 2.2. Quantitative Analysis of ARGs and intl1

**Figure 2:**
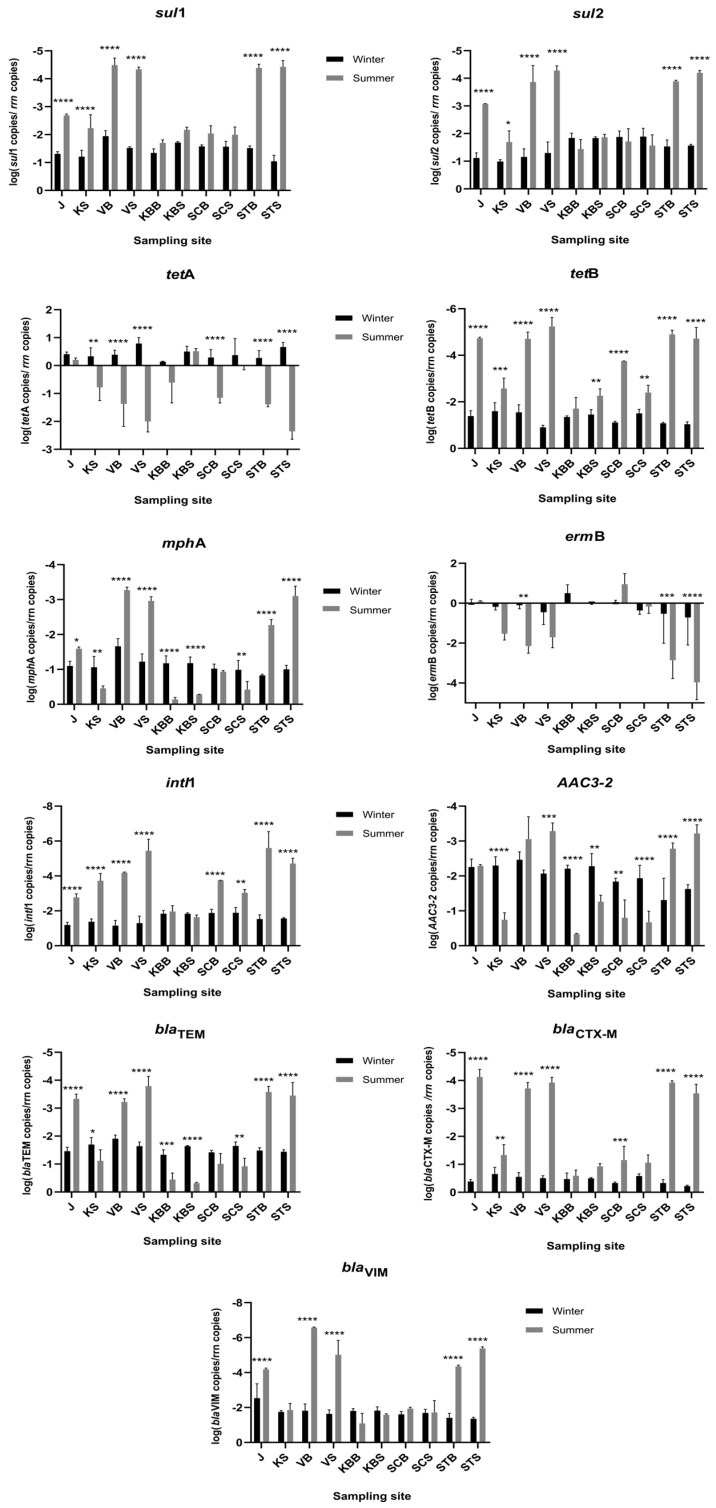
Seasonal and spatial distribution of targeted genes along the trophic gradient represented by their relative abundance (copies of gene/16S rRNA gene). Data were compared using two-way analysis of variance (ANOVA), and seasonal significance level is represented by the *p*-value (Sidak test for multiple comparisons). **** *p* < 0.0001; *** *p* < 0.005; ** *p* < 0.001; * *p* < 0.01.

The abundance of ten ARGs (*sul1*, *sul2*, *tetA*, *tetB*, *mphA*, *ermB*, *aac3-2*, *bla*_TEM_, *bla*_CTX-M,_ *bla*_VIM_) and the integrase gene *intl1* was determined using a qPCR approach. All genes were detected at all sites along the trophic gradient, but their relative abundance varied spatially and seasonally (up to 10,000-fold) (Figure 2; Table S3). Overall, the most frequently detected ARGs were the macrolide resistance gene *ermB* and the tetracycline resistance gene *tetA*, irrespective of season and trophic status. The gene *ermB* was most abundant in the lower layer of the oligotrophic SCB in summer (8.36 copies/*rrn*), while *tetA* was most abundant in the eutrophic VS in winter (6.64 copies/*rrn*). Interestingly, the macrolide resistance-related gene *mphA* was also among the most frequently detected genes along the trophic gradient. The gene *mphA* was more abundant in the coastal area in summer (1.02, 0.7, and 0.3 copies/*rrn* in KBB, SCS, and KS), while it was most frequent in the oligotrophic area and the open sea in winter (0.14, 0.10, 0.11, and 0.09 copies/*rrn* in STB, STS, SCS, and SCB), respectively.

The abundance of the β-lactam resistance genes (*bla*_TEM_, *bla*_CTX-M_, *bla*_VIM_) showed no clear spatial and temporal character. In particular, *bla*_TEM_ (detected in a range from 2 × 10^−4^ in STB to 0.87 copies/*rrn* in KBB) and *bla*_VIM_ (4 × 10^−6^ in STS to 0.14 copies/*rrn* in KBB) showed seasonality and were generally found more frequently in summer than in winter, following the trophic gradient. In contrast, *bla*_CTX-M_ is most abundant in winter (from 0.19 copies/*rrn* at SCS to 0.6 copies/*rrn* at oligotrophic open-sea STS in winter) and was not related to spatial status.

The relative abundance of sulfonamide resistance genes *sul1* and *sul2* appears to be site-specific, although both were uniformly more abundant in winter. Comparing their abundance with each other, *sul2* was generally more abundant along the trophic gradient regardless of season (maximum 0.14 copies/*rrn* in winter and up to 0.04 in summer at KBB).

The relative abundance of the integrase gene *intl1* steadily followed the trophic gradient and occurred more frequently in winter (maximum 0.08 copies/*rrn* at VB) than in summer, when it shifted to the central part of the bay (KBS 0.02 copies/*rrn* at KBS). Moreover, the aminoglycoside resistance gene *aac3-2* was the least abundant ARG across the trophic gradient, with no obvious spatial shifts, while its seasonal variation (up to tenfold) was statistically significant at almost all sites (Figure 2; Table S3).

Taken together, the coastal eutrophic sites (J, KS) showed significant seasonal shifts (*p* < 0.0001) for all genes except *mphA*, *ermB*, *aac3-2*, and *tetA.* The mesotrophic area (KBB, KBS, SCS, and SCB) was seasonally stable, with the exception of *bla*_TEM_, *tetB*, *aac3-2*, *mphA*, and *intl1*, which were significantly more abundant in winter (*p* < [0.0001–0.0017]).

### 2.3. Seasonal and Spatial Co-Occurrence of Quantified ARGs and intl1

**Figure 3:**
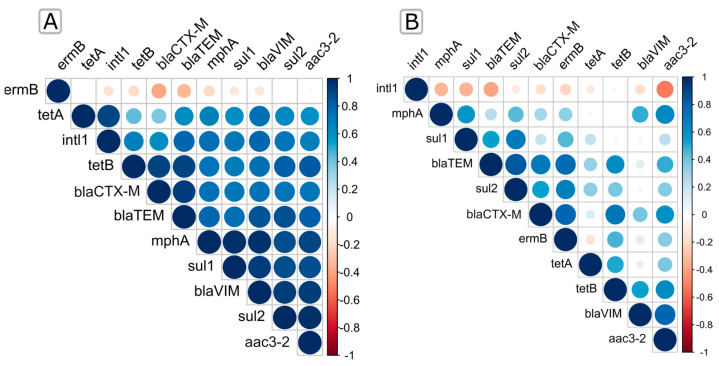
Spearman’s rank correlations of the ARGS relative abundances during summer (**A**) and winter (**B**). Positive correlations are displayed in blue, and negative correlations are displayed in red. Color intensity and the size of the circles are proportional to the correlation coefficients.

**Figure 4:**
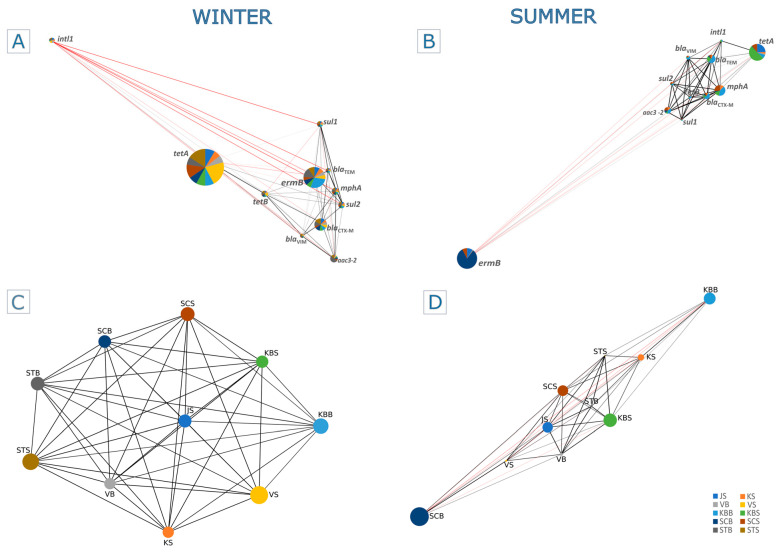
Mentel–Pearson correlation of ARGs distribution along the trophic gradient. The co-occurrence network represents the relations between the ARGs, where the node size is proportional to the abundance of the gene in winter (**A**) and summer (**B**). Sampling sites are represented by different colors, and their distribution within each node is defined by the frequency of a specific gene at that site. The Mentel–Pearson correlation between the sampling sites is shown for winter (**C**) and summer (**D**), with a positive correlation represented by a black line and a negative correlation by a red line.

The relationship between the targeted ARGs along the trophic gradient in the summer and winter seasons was represented by the Spearman rank correlation heatmap (Figure 3; Table S4) and the Pearson correlation-based co-occurrence network (Figure 4; Table S5).

We found that *ermB* in summer is the only gene negatively correlated with all compared genes except *aac3-2* (Spearman *r* < 0.03), while all other genes were negatively correlated with at least one gene (Figure 3A; Table S4), as also confirmed by the Pearson correlation (Figure 4B). In addition, we found that *sul1*, *sul2*, *bla*_TEM_, and *bla*_VIM_ showed a significant positive correlation (Spearman *r* > 0.79, *p* < 0.0001) with *mphA.* A positive correlation was also observed between *bla*_CTX-M_, *bla*_TEM_, and *tetB* (Spearman *r* > 0.92, *p* < 0.0003) (Figure 3A; Table S4), which was also confirmed by the Pearson correlation (*r* = 0.97 and 0.99) (Figure 4B; Table S5). The integrase gene *intl1* most significantly correlated with *tetA* (Spearman *r* > 0.9, *p* < 0.0003; Pearson *r* = 0.82), *bla*_TEM_, and *bla*_VIM_ (Spearman *r* > 0.8, *p* < 0.006; Pearson *r* = 0.75 and 0.45), followed by other ARGs (*p* < 0.05), while it only exhibited a negative correlation only with *ermB* (Spearman *r* < −0.16; Pearson *r* = −0.2) (Figure 3A and Table S3; Figure 4B and Table S5).

During winter, the frequencies of ARGs showed very different relations, indicating the considerable influence of seasonality (Figure 3B and Table S4; Figure 4A and Table S5). For instance, the relative abundance of *bla*_TEM_ showed a significant positive correlation (Spearman *r* > 0.83, *p* < 0.002) with *sul2* and *bla*_CTX-M_, while it was negatively correlated with *intl1* (Spearman *r* < −0.39). A positive and statistically significant Spearman correlation was also observed between *ermB* and the β-lactam resistance genes *bla*_CTX-M_ and *bla*_TEM_, as shown by the Pearson coincidence network. In contrast, the integrase gene *intl1* showed a negative Spearman correlation with all genes examined in winter (Figure 3B; Table S4).

In addition, the Spearman correlation showed a strong linear relationship between trophically similar sites in winter (Figure 4C), with eutrophic/mesotrophic sites clearly grouped against oligotrophic sites. However, this was less clear in summer (Figure 4D), as the high abundances of *ermB* and *tetA* genes led to a decrease in the linear relationship between these areas.

### 2.4. Relationship between Environmental Parameters and Gene Abundances

**Figure 5:**
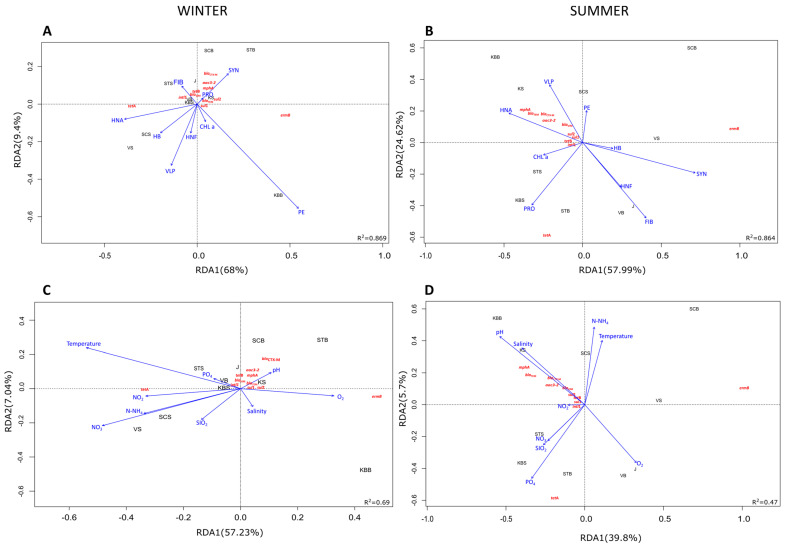
Redundancy analysis (RDA) ordination plot illustrating the correlation between the ARGs and biological properties (**A**,**B**) or physicochemical properties in winter (**C**) and summer (**D**). Abbreviations: temperature (TEMP), salinity (SAL), pH, dissolved inorganic nutrients (NO^3−^, NO_2_^−^, NH_4_^+^, and PO_4_^3−^), dissolved oxygen (O_2_), chlorophyll *a* (Chl *a*), *Synechococcus* (SYN), *Prochlorococcus* (PRO), picoeukaryotes (PE), heterotrophic bacteria (HB), heterotrophic nanoflagellates (HNF), virus-like particles (VLP), fecal indicator bacteria (FIB).

The relationship between environmental factors, ARG, and sampling sites was examined using RDA (Figure 5). Taken together, the environmental variables explained a total of 99.8% of the variation in ARG abundance along the trophic gradient. During winter, the cyanobacteria SYN and PRO acted synergistically and had a strong positive effect on the distribution of most ARGs along the first canonical axis (Figure 5A). In contrast, HNA, HNF, HB, and VLP were negatively correlated with most genes, with the exception of *tetA* at mesotrophic (VS) and oligotrophic (SCS) sites and *ermB* at the eutrophic KBB site. It is noteworthy that the abundance of *intl1* correlated negatively with PE and Chl *a* and positively with FIB along the trophic gradient (STS, VB, and KBS). In terms of physico-chemical parameters, pH was found to have a positive influence on the distribution and abundance of ARGs in the eutrophic coastal zone (KS, J) in winter (Figure 5C). In the oligotrophic zone (STS, SCS), the main driving factors with positive correlation were dissolved inorganic nutrients (NO_3_^−^, NO_2_^−^, NH_4_^+^), while SAL had a negative effect. TEMP was found to have a negative effect on the abundance of ARGs (R^2^ = 0.69, *p* < 0.034), except in the case of *tetA*, whose abundance was positively affected, especially in the mesotrophic zone (VS). The abundance of *ermB* also showed a very strong positive correlation with SAL and dissolved oxygen in this zone (KBB) (Figure 5C).

Regarding the summer season, the RDA revealed that HNA and VLP were the main biological factors positively influencing the distribution of all ARGs, except *tetA* and *ermB*, along the trophic gradient. The gene *tetA* was found to be positively correlated only with Chl *a* and PRO in mesotrophic and oligotrophic areas (KBS, STS, STB). However, the substantial influence of HB, HNF, SYN, and FIB at eutrophic (J) and mesotrophic sites (VB) did not affect the abundance of target ARGs in summer (Figure 5B). Moreover, variations in pH and SAL were positively correlated with the abundance of all genes except *tetA* along the trophic gradient, while this ARG was positively affected by some inorganic nutrients (NO_3_^−^ and PO_4_^3−^) (Figure 5D).

### 2.5. Association of Quantified ARGs with Bacterial Communities along the Trophic Gradient

**Figure 6:**
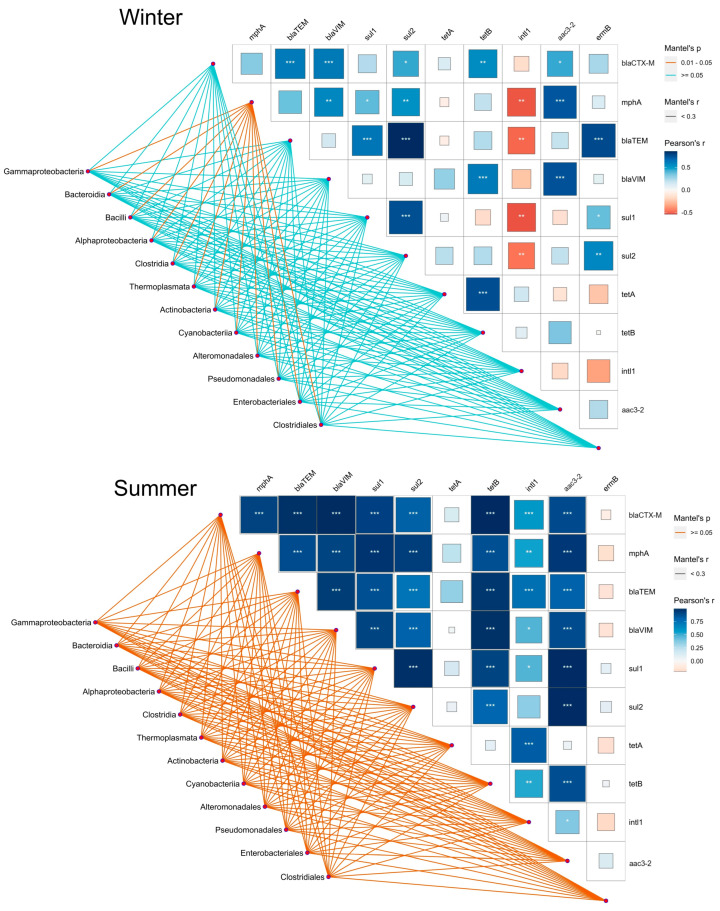
Pairwise comparisons of ARGs and their association with the selected bacterial classes and orders are shown with a color gradient denoting Pearson correlation coefficient. The eight most abundant classes and four orders were selected to provide a better insight seasonally into the differences between eutrophic and oligotrophic sites. The bacterial classes and orders were related to each ARG based on their abundance. The edge width corresponds to the values of Mentel’s *r*, and the line color indicates the statistical significance of Mentel’s *p* value. *** *p* < 0.005; ** *p* < 0.001; * *p* < 0.01.

To investigate the relationships between the ARG profiles obtained in this study and the bacterial communities previously analyzed by Illumina-based metagenomics [8], we performed a Mentel–Pearson correlation by taking the most abundant bacterial classes along the trophic gradient during summer and winter (Figure 6).

In winter, the only statistically significant (*p* < 0.01) relation of classes, including *Gammaproteobacteria*, *Bacilli*, and *Bacteroidia*, and genes were shown for *mphA*, which further positively correlated with *aac3-2*, *bla*_VIM_, and *sul2* and negatively correlated with *intl1*. The gene *intl1* displayed a negative correlation with a number of other genes, of which the *sul*-like genes and *bla*_TEM_ were found to be significant for all studied orders, such as *Pseudomonadales*, *Enterobacteriales*, and *Clostridiales*. On the other hand, a positive but not statistically significant interconnection was observed between bacterial classes and ARGs during the summer. In comparison to winter, *mphA* and *bla*_TEM_ correlated positively when compared to *tetA* and associated with the aforementioned classes, while this was even more evident in the case of the *intl1* gene. On the contrary, the abundance of *ermB* in bacterial classes and classes during summer shifted to a negative correlation when compared to the *mphA* and *bla* genes (Figure 6).

## 3. Discussion

By combining culture-dependent and culture-independent techniques, including qPCR in this study and the Illumina-based amplicon sequencing in our previous study [8], we further investigated the distribution of clinically relevant ARGs along the trophic gradient of the Adriatic Sea to obtain a more comprehensive picture of how ARGs disperse, which particular environmental parameters influence them the most, and which bacterial taxa may be their key mediators in the temperate marine environment.

### 3.1. Seasonality Is a Driving Factor for Antibiotic Resistance and Distribution of ARGs

Heterotrophic bacterial counts revealed that the incidence of resistance to some antibiotics differed significantly when comparing the remote oligotrophic areas with the human-influenced coastal areas, with the incidence generally higher in winter than in the summer.

This could be related to the fact that we have previously observed higher abundances of human gut-associated bacteria in coastal waters in winter as the seawater remains closer to the sewage outlets due to the coastal relief and winds [13]. Less activity of predators (HNF) and weaker insolation and surface temperature in winter could increase the bacterial survival rate compared to summer [14]. In addition, we have already observed that offshore sites are characterized by a shift of ARGs towards the intrinsic resistance mechanisms of autochthonous marine bacteria and are strongly affected by seasonality, which is primarily related to the stratification of the water column in summer [8].

The prevalence of resistance to TET was among the highest across the trophic gradient, although it decreased away from the coast and was lowest in oligotrophic waters. This suggests that it is an anthropogenic feature, as the coastal waters serve as recipients of sewage discharges and effluents from fish farms. Namely, as a broad-spectrum antibiotic, TET serves as a growth promoter in aquaculture [15] and is a widely used antimicrobial in human medicine, which is excreted into the environment in an active form via urine and feces after treatment [16]. Accordingly, *tetA* was found to be the most abundant ARG in winter and the second most abundant in summer along the trophic gradient. An analysis of the global ocean resistome based on data from the TARA Oceans project found *tetA* and *tetB* to be among the most abundant oceanic ARGs [17]. Regarding the surface–bottom layer distribution, TET resistance was more abundant in surface waters in winter, while the bottom and surface layers were somewhat similar in the summer season (Figure 1). Warmer temperatures and higher nutrient loads in the sea, e.g., in estuaries and from sewage in coastal areas, are crucial factors for the growth and dynamics of bacterial communities [4] and affect the survival and distribution of bacteria associated with sewage, leading to a shift in bacterial gene profiles [18]. In our previous study [8], PICRUSt2 functional prediction based on the 16S rRNA amplicon sequencing data showed that the *tetA* and *tetB* genes were highly represented in marine microbial communities of the eutrophic and mesotrophic areas in winter.

The same analysis indicated a higher occurrence of sulfonamide resistance genes *sul1* in the estuary and *sul2* in the bottom layer of the oligotrophic zone [8], which was confirmed by qPCR experiments in this study. *Sul* genes are among the most abundant ARGs in surface marine waters [19], especially in beach waters [20] as well as in rivers [21]. In addition, they generally appear to be more recalcitrant and have a higher potential to increase in abundance than other ARGs in the environment [22,23]. Accordingly, a high relative abundance of these genes was found in eutrophic and mesotrophic zones, while they showed seasonal stability in both the surface and bottom layers of the water column.

As for the prevalence of beta-lactam (CTX) resistance, its seasonal variations reflected the distribution of the *bla*_CTX-M_ gene along the trophic gradient, which was more abundant in winter at both mesotrophic and oligotrophic sites. In addition, the high relative abundance of the *bla*_TEM_ gene (up to 0.87 copies/*rrn* at KBB) corroborates the finding that beta-lactam resistance is highest in coastal waters [20,24], where it correlates strongly with the respective anthropogenic influences [25]. However, the aminoglycoside resistance gene *aac3-2* was the least abundant ARG across the trophic gradient in this study, with no apparent increase, although a relatively higher abundance of this gene had previously been found in the anthropogenically impacted coastal areas [26].

The gene *intl1* had been previously used as an indicator of anthropogenic activities [25], while integrons as mobile genetic elements are mediators of horizontal gene transfer and, thus, important factors for the widespread distribution of ARGs in the environment [27,28]. Consistent with this, the distribution of *intl1* steadily followed the trophic gradient and was more abundant in winter, as were the majority of ARGs analyzed in this study. In summer, relative abundance was particularly high in the mesotrophic area. Nevertheless, previous studies have shown that the scenario in the coastal environment is very complex due to the various external sources of ARGs [3,29].

### 3.2. Correlation between ARGs, intl1, and Physico-Chemical Parameters Seasonally

In this study, most ARGs showed significant seasonal variation, with relatively higher relative abundance in winter. It is noteworthy that the relative abundance of *intl1* correlated negatively with PE and Chl *a* and positively with FIB along the trophic gradient, indicating *intl1* as a reliable marker of anthropogenic influence in the natural environment. Furthermore, the positive correlation of *intl1* with the majority of clinically important ARGs analyzed in this study, especially the *bla*, *tetA*, and *mphA* genes, underscores the relationships between the anthropogenic impact and the distribution of ARGs. The significant correlation between *tet* genes and *intI1* has also been frequently observed in various aquatic environments [23], where it plays a key role in the integration of exogenous ARGs into the bacterial genome [30,31]. These co-occurring ARGs are often associated with integrons, transposons, or plasmids that enable the transfer of ARGs between different bacterial taxa [32]. In this context, several genetic determinants of antimicrobial resistance, such as *bla*_TEM_, *sul1*, and *intI1*, have already been proposed as valuable indicators of contamination to assess the level of antimicrobial resistance in the environment [33]. In addition, a high concentration of organic and inorganic matter introduced by submarine sewage effluents [6] or river inflows stimulates the growth of bacterioplankton [34] and virio-plankton, as the abundance of viruses is closely correlated with the abundance of bacteria [35]. Bourdonnais et al. [36] investigated the abundance of ARGs in the English Channel (North Sea) and found that the *sul1* and *intI1* genes were positively correlated with dissolved oxygen, while the microbial population was also correlated with pH, TEMP, and SAL in addition to dissolved oxygen and turbidity.

In the oligotrophic zone, the main driving factors with positive correlation were dissolved inorganic nutrients, suggesting that nutrient loading may further promote the maintenance of ARGs in the natural aquatic environment [37]. Environmental stresses such as extreme temperatures, pH, and SAL may induce bacteria to cope with these stresses through phenotypic and genotypic adaptations that enable subsequent resistance to similar stresses [38]. Indeed, TEMP was found to have a negative effect on the distribution of all analyzed ARGs except *tetA* in the mesotrophic zone. Moreover, the abundance of *ermB* showed a strong positive correlation with SAL and dissolved oxygen in the mesotrophic zone in winter. In summer, SAL was positively correlated with all ARGs except *tetA*, whose abundance was positively influenced by inorganic nutrients, as previously shown [39]. Conversely, the distribution of *tetA* at mesotrophic and oligotrophic sites in winter was mainly influenced by HNA, HNF, HB, and VLP. Previously, Ordulj et al. [40] found the lowest virus abundances in summer at coastal sites in the central and southern Adriatic.

The macrolide gene *ermB* correlated positively only with *aac3-2* in summer but with *bla*_CTX-M_ and *bla*_TEM_ in winter. Regarding the biological parameters, *ermB* showed a positive correlation with HNA, HNF, HB, and VLP at eutrophic sites in winter and an opposite correlation in summer. Notably, the distribution of *ermB* did not follow the trophic gradient and also showed no positive correlation with the nutrient/FIB increase, so it cannot be considered a biomarker for tracking antibiotic resistance.

### 3.3. Association of Quantified ARGs with Bacterial Communities along the Trophic Gradient

The co-occurrence network analysis revealed a significant association of the ARGs quantified in this study with the most abundant bacterial classes along the trophic gradient. A significant relationship between the bacterial populations and the ARGs was observed in both marine [41] and estuarine environments [42]. The *intI1* gene in pollution-impacted surface waters was found to be distributed mainly between *Gammaproteobacteria*, such as clinically relevant families of *Enterobacteriaceae, Pseudomonadaceae*, and *Aeromonadaceae*, and co-occured with genes encoding beta-lactamase, sulfonamide, aminoglycoside, and tetracycline resistance [43]. The *sul1*, *sul2*, and *tetA* are commonly found with *intl1* in broad host range plasmids, contributing to the spread of multiresistance in aquatic environments [44]. *Enterobacteriaceae*, particularly *E. coli*, *Enterobacter* spp., and *Citrobacter* spp., can simultaneously carry these resistance determinants [45,46].

Dželalija et al. [8] reported that *tetA* was associated with *Enterobacterales* (*Proteus* spp.), *Gammaproteobacteria* (*Vibro* spp.), and *Bacilli* (*Bacillus* spp.), increasingly in the winter season, in eutrophic and mesotrophic areas. In this study, a higher positive correlation was found with these taxa and, furthermore, with *Bacteroidia, Pseudomonadales*, and *Clostridiales.* The seasonal shift of *tet* genes toward a higher relative abundance in winter could be related to sewage discharges and runoff from the mainland, as speculated previously [8].

Furthermore, we have already predicted that *sul* genes are associated with a high abundance of *Gammaproteobacteria* in the eutrophic zone, especially in the estuary in winter, and are carried by *Acinetobacter*, *Enterobacter*, and *Aeromonas* further along the trophic gradient [8]. The detection of these genes in high relative abundances by qPCR in this study, thus, provides further evidence of their persistence in the human-impacted aquatic environment, where *Protebacteria* (mainly *Gammaproteobacteria*) are the predominant carriers of ARGs [19,47,48]. Beta-lactam-encoding genes (*bla*_TEM_ and *bla*_VIM_) were detected more frequently in summer, especially in eutrophic areas, which could be associated with a high abundance of *Vibrio*, *Acinetobacter*, and *Citrobacter* as their probable carriers [8]. On the other hand, the abundance of *bla*_CTX-M_ did not decrease away from the coast, which could be explained by the shift of the community from *Gammaproteobacteria* in winter to Marine group II in summer at mesotrophic and oligotrophic sites [8].

The macrolide resistance genes *ermB* and *mphA,* which were among the most frequently detected ARGs, may be related to *Acinetobacter*, *Bacteroidetes*, and *Bacillus*, all of which are highly abundant in coastal marine microbiomes [8]. Consistent with the results of our study, Li et al. [19] reported that *Bacteroidetes* are positively correlated with several ARGs in the marine environment, while *Proteobacteria* are correlated with *intl1* [49].

## 4. Materials and Methods

### 4.1. Study Area and Determination of Environmental and Biological Parameters

**Figure 7:**
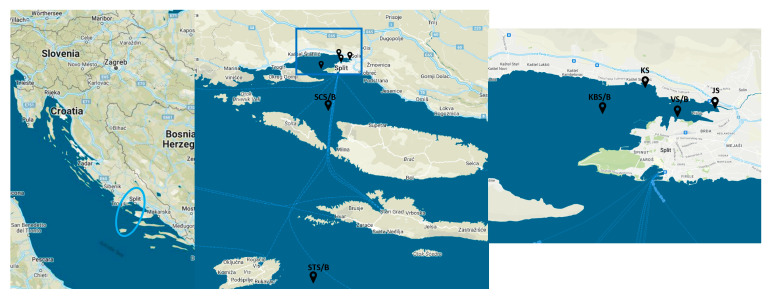
Map of the study area and sampling sites in the central Adriatic Sea. JS, Jadro River; KS, Kaštel Sućurac; VB, Vranjic Bay (bottom layer); VS, Vranjic Bay (surface layer); KBB, Kaštela Bay (bottom); KBS, Kaštela Bay (surface); SCB, Split Channel (bottom); SCS, Split Channel (surface); STB, Stončica (bottom); STS, Stončica (surface).

Water samples from the bottom (extension B in sample name) and surface (extension S) layers were collected seasonally in March (winter) and August (summer) 2021 in the central Adriatic Sea (Croatia), as previously described [8] (Table S1). The six sampling sites were chosen based on geography and anthropogenic impact along the trophic gradient over a 68 km line from the wastewater-influenced estuary to coastal area and the pristine open sea (Figure 7). The eutrophic JS represents the site within the Jadro River estuary affected by unmanaged wastewater from households [5]. The river further flows into the Vranjic Bay, represented by eutrophic coastal VS/VB site. The following KSS site is located near a wastewater-impacted public beach on the coast of the nearby Kaštela Bay [13] and is also eutrophic. KBS/KBB site is mesotrophic and located in the center of Kaštela Bay but is still influenced by the mainland, while the offshore oligotrophic station SCS/SCB further follows the trophic gradient. The last sampling site, an open-sea STS/STB, is located near the island of Vis, one of the most remote islands in the eastern Adriatic, and is considered a pristine marine environment.

Physico-chemical and biological parameters were determined previously, and the data and standard protocols were given previously [8] (Table S1). These included the following: temperature (TEMP), salinity (SAL), pH, dissolved inorganic nutrients (NO_3_^−^, NO_2_^−^, NH_4_^+^, and PO_4_^3−^), dissolved oxygen (O_2_), chlorophyll *a* (Chl *a*), abundances of *Synechococcus* (SYN), *Prochlorococcus* (PRO), picoeukaryotes (PE), heterotrophic bacteria (HB), heterotrophic nanoflagellates (HNF), and viruses, as well as bacterial production and enumeration of fecal indicator bacteria (FIB) (*E. coli* and intestinal enterococci).

### 4.2. Antibiotic-Resistant Heterotrophic Bacterial Counts

Aliquots (100 µL and 1 mL) of seawater were plated on Marine agar (MA; BD Difco, Franklin Lakes, NJ, USA) plates without antibiotics, while 10, 25, 50, and 100 mL of seawater were filtered using 0.22 µm pore-size mixed cellulose ester (MCE) membranes (Whatman, Maidstone, UK), which were placed on MA plates supplemented with an appropriate antibiotic from the β-lactam, sulfonamide, macrolide, tetracycline, or aminoglycoside class to enumerate resistant and total heterotrophic bacteria. Based on the minimal inhibitory concentration (MIC) breakpoint interpretive for antibiotic resistance provided by Clinical and Laboratory Standards Institute [50] and The European Committee on Antimicrobial Susceptibility Testing [51], the plates were supplemented with 32 µg/mL of azithromycin (AZM), 4 µg/mL of cefotaxime (CTX), 16 µg/mL of gentamicin (GEN), 512 µg/mL of sulfamethoxazole (SXT), and 16 µg/mL of tetracycline (TET). Experiments were performed in triplicates. Colonies were counted after the incubation at 28 °C for 7 days, and the frequency (%) of antibiotic-resistant bacteria (i.e., number of antibiotic-resistant colony-forming units (CFU) per ml of water divided by total CFU, multiplied by 100) was determined for each sample. All antibiotics were purchased in the form of powder from Sigma-Aldrich (St. Louis, MO, USA).

### 4.3. DNA Extraction and Quantification of Target Genes

Two liters of water were filtered in triplicate using fast-flow 0.22 µm pore-size MCE membranes (Whatman), after which the genomic DNA was extracted using PowerWater DNA Isolation Kit (Qiagen, Hilden, Germany) following the manufacturer’s instructions. DNA concentration was measured using NanoDrop^®^ spectrophotometer ND-1000 (Thermo Fisher Scientific, Waltham, MA, USA), and DNA was stored at −20 °C for further analysis.

Quantitative real-time PCR (RT-qPCR) was used to quantify the 16S rRNA gene, class 1 integron-integrase gene (*intl1*), and ten ARGs, including sulfonamide resistance genes (*sul1*, *sul2*), tetracycline resistance genes (*tetA, tetB*), β-lactam resistance genes (*bla*_CTX-M_, *bla*_TEM_, *bla*_VIM_), macrolide resistance genes (*ermB*, *mphA*), and aminoglycoside resistance gene (*aac3-2* encompassing *aac(3′)-IIa/aacC3/aacC2* and *aac(3′)-IIc/aacC2*) using primers and amplification conditions listed in Supplemental Table S6. The specificity of all primers was confirmed by solubility curve analysis. Genes of interest were first amplified from the genomic DNA of antibiotic-resistant bacteria, separated by standard gel electrophoresis, purified using ReliaPrep™ RNA Clean-Up and Concentration System (Promega, Southampton, UK), and then cloned into pGEM^®^-T Easy Vector or pNORM plasmids (Promega) according to the manufacturer’s recommendations. The recombinant plasmids were then transformed into competent *E. coli* JM109 (Promega). After positive clones were selected and verified by PCR, the plasmids were used to prepare qPCR standards. Each qPCR reaction was performed in a total volume of 20 μL using 10 μL Power SYBR^®^ Green PCR Master Mix (Applied Biosystems, Waltham, MA, USA), 1 μM of each primer, and 2 ng of DNA template in 96-well plates. All assays were carried out on the ABI 7500 Fast Real-time PCR System (Applied Biosystems). The efficiency and sensitivity of each assay were determined by generating a standard curve using 10-fold serial dilutions of plasmid DNA. Reliable correlation coefficients (R^2^ > 0.99) and amplification efficiencies based on slopes between 98% and 105% for standard curves were obtained. Melting curves were generated to confirm amplification specificity. All qPCR assays were performed using three biological replicates and two technical replicates, whereas a non-template sample (containing DNA-free water) was included as a negative control.

### 4.4. Data Analysis and Statistics

RT-qPCR data were given as the relative abundance of the target gene in each sample, calculated as the ratio of ARG or *intl1*/16S rRNA gene copy number (*rrn*), log10 transformed and subjected to a Shapiro–Wilk test to assess their normal distribution. Data were then compared using a two-way analysis of variance (ANOVA). Sidak’s multiple comparisons test was performed to compare the relative abundance of genes at each site between two seasons and visualized using GraphPad Prism V9.01. Statistically significant differences in gene abundances and environmental parameters were evaluated using the program R (v3.4.2) and redundancy analysis (RDA) using the packages vegan, ggplot2, labdsv, MASS, and mvpart. Gene frequencies were normalized using the Hellinger transformation, and environmental parameters were standardized. To investigate the correlation effect of ARGs mutually, a Spearman rank correlation test was performed, and correlation matrices were developed. The statistical analysis was performed with the software Rstudio (v2023.09.1 + 494) using the packages agricolae, stats, and corrplot.

In our previous study [8], we conducted a comprehensive metagenomic analysis. In brief, 16S rRNA amplicon sequencing of the V3-V4 hypervariable region was performed. The 250 bp paired-end raw reads were merged, and high-quality clean tags were obtained after quality filtering with QIIME2 (v2022.2). Sequences with ≥97% similarity were assigned to the same Operational Taxonomic Units (OTUs) by the Closed Reference clustering method. The OTU sequences were classified using the vsearch method and the SILVA database (v.13.8) to determine relative taxa frequencies and construct a phylogenetic tree. Diversity analysis and functional prediction were performed using QIIME2 (v2022.2) and PICRUSt2 on the Nephele platform [52,53]. Filtering of metagenomic data for visualization was performed using the in-house program MiaViz (https://github.com/apavlinovic/mia-viz; accessed on 22 September 2023) and Python 3.9.0 (library: Pandas, SciPy, Seaborn).

The co-occurrence network was constructed to show the correlations between the genes (Pearson’s correlation) using the cooccure package in R. All statistical tests were considered significant at *p* < 0.05. Mantel test and Pearson’s correlation were applied to assess the relationships between the bacterial communities at the class level and the abundances of ARGs using the ggcor package in R.

## 5. Conclusions

In the present study, the distribution of ARGs denoting resistance to five major antibiotic classes was characterized in the central Adriatic by combining culture-dependent and culture-independent techniques, including RT-qPCR and metagenomic sequencing, which provided further insights into the profiles of ARGs and specific bacterial taxa as their carriers in the marine environment. As also revealed by metagenomics, a higher resistance was detected by qPCR in the winter eutrophic zone, which was due to the high relative abundance of *tetA*, *bla*_CTX-M_, and *bla*_TEM_ genes associated with the higher TEMP, higher nutrient loading, and lower SAL, as well as the high abundance of *Gammaproteobacteria* such as *Acinetobacter* and *Citrobacter*. A different trend was observed in the oligotrophic zone, where *tetA* was mainly driven by TEMP and HNF, while dissolved oxygen and SAL were responsible for *ermB.* In addition, *intl1* steadily followed the trophic gradient, positively correlated with FIB, and was more abundant in winter, suggesting that this gene is a favorable gene marker for tracking anthropogenic impacts in the natural environment. A similar pattern was also observed for *bla*_TEM_ and *bla*_VIM_. Water column stratification and geography significantly influence ARGs distribution, favoring eutrophic, especially estuarine, areas, while seasonal changes in oligotrophic sites result from a shift in bacterial communities from *Gammaproteobacteria* in winter to Marine group II in summer.

## Data Availability

Data are contained within the article and supplementary materials.

## Supplementary Materials

The following supporting information can be downloaded at: https://www.mdpi.com/article/10.3390/ijms25010654/s1.
